# Altered Synaptic Membrane Retrieval after Strong Stimulation of Cerebellar Granule Neurons in Cyclic GMP-Dependent Protein Kinase II (cGKII) Knockout Mice

**DOI:** 10.3390/ijms18112281

**Published:** 2017-10-30

**Authors:** Andrea Collado-Alsina, Franz Hofmann, José Sánchez-Prieto, Magdalena Torres

**Affiliations:** 1 Departamento de Bioquímica, Facultad de Veterinaria, Universidad Complutense, 28040 Madrid, Spain; andrea.collado@pdi.ucm.es (A.C.-A.); jsprieto@ucm.es (J.S.-P.); 2 Instituto de Investigación Sanitaria del Hospital Clínico San Carlos (IdISSC), 28040 Madrid, Spain; 3 FOR 923, Institut für Pharmakologie und Toxikologie, Technische Universität München, Biedersteiner Str. 29, 80802 Munich, Germany; Franz.Hofmann@mytum.de

**Keywords:** cerebellar granule cells, cGKI, cGKII, synaptic vesicle recycling, FM1-43, KT5823, VGluT-pH

## Abstract

The nitric oxide (NO)/cyclic guanosine monophosphate (cGMP)/cGMP-dependent protein kinase (cGK) signaling pathway regulates the clustering and the recruitment of proteins and vesicles to the synapse, thereby adjusting the exoendocytic cycle to the intensity of activity. Accordingly, this pathway can accelerate endocytosis following large-scale exocytosis, and pre-synaptic cGK type II (cGKII) plays a major role in this process, controlling the homeostatic balance of vesicle exocytosis and endocytosis. We have studied synaptic vesicle recycling in cerebellar granule cells from mice lacking cGKII under strong and sustained stimulation, combining imaging techniques and ultrastructural analyses. The ultrastructure of synapses in the adult mouse cerebellar cortex was also examined in these animals. The lack of cGKII provokes structural changes to synapses in cultured cells and in the cerebellar cortex. Moreover, endocytosis is slowed down in a subset of boutons in these cells when they are stimulated strongly. In addition, from the results obtained with the selective inhibitor of cGKs, KT5823, it can be concluded that cGKI also regulates some aspects of vesicle cycling. Overall, these results confirm the importance of the cGMP pathway in the regulation of vesicle cycling following strong stimulation of cerebellar granule cells.

## 1. Introduction

Both nitric oxide (NO) and its downstream messenger, cGMP, have been implicated in the development of the nervous system, from neurogenesis and neuron migration to synaptogenesis [1,2,3]. During synaptic transmission, NO forms as a consequence of *N*-methyl-d-aspartic acid receptor (NMDAR) stimulation at glutamatergic synapses. Synaptic transmission is not strictly unidirectional and a variety of feedback signals flow from postsynaptic sources towards presynaptic targets, NO being one such signal [4]. Indeed, the NO-activated cGMP pathway is essential to coordinate functional and structural alterations at pre- and postsynaptic sites in response to neuronal activity [5,6].

At postsynaptic sites, cGKII phosphorylates GluA1 at S845 and it augments the surface expression of calcium permeable α-amino-3-hydroxy-5-methyl-4-isoxazolepropionic acid receptors (AMPARs) at extrasynaptic sites. This signaling lies downstream of NMDA receptor activation, and is likely to be relevant for the development and plasticity of both cerebellar and hippocampal neurons [7,8]. At presynaptic sites, the NO/cGMP pathway regulates the clustering and recruitment of synaptic proteins and vesicles [9], and it adjusts the rate of the exoendocytic cycle to the intensity of activity [10,11,12,13]. These presynaptic effects are mainly mediated by the activation of cGMP-dependent protein kinases (cGKs) [5,14].

There are abundant cGKII transcripts in the brain [15], and a mouse model that lacks this kinase exhibits deficits in spatial learning and impaired working memory [16], both of which could be explained by synaptic dysfunction. Although a network that compensates for the loss of cGKII has been described in the hippocampus to regulate GluA1 phosphorylation [17], other synaptic alterations might explain the deficits observed in cGKII knock out (KO) mice. We previously demonstrated that the blockage of NMDAR [18] or the inhibition of soluble guanylyl cyclase (sGC) [3] impaired synaptic vesicle (SV) recycling in rat cerebellar granule cells during strong and sustained stimulation. In addition, we showed that these effects were prevented by pharmacologically increasing cGMP and that they were mimicked by inhibiting or silencing cGKII. Hence, we have now analyzed this process in cells from cGKII KO mice using imaging techniques (FM1-43 and vGluT1-pH), ultrastructural analyses and pharmacological inhibition of cGKs in order to study SV recycling in these cultured neurons and to evaluate the involvement of cGKI in its regulation. The lack of cGKII provokes structural changes at synapses in either cultured cells or cerebellar cortex. Although cGKII KO neurons do not fully reproduce the effects of acute inhibition of this protein in control cells, probably due to functional compensation, membrane retrieval is altered in these cells when they are stimulated strongly, confirming the importance of cGKII in controlling the homeostatic balance of vesicle exocytosis and endocytosis at synapses in cerebellar granule cells. Moreover, by using a selective inhibitor of cGKs, KT5823, in cGKII KO neurons has allowed us to conclude that cGKI is also involved in the regulation of vesicular cycle.

## 2. Results

### 2.1. Efficient SV Recycling in Boutons from Mice Cerebellar Granule Cells after Strong and Sustained Stimulation

**Figure 1:**
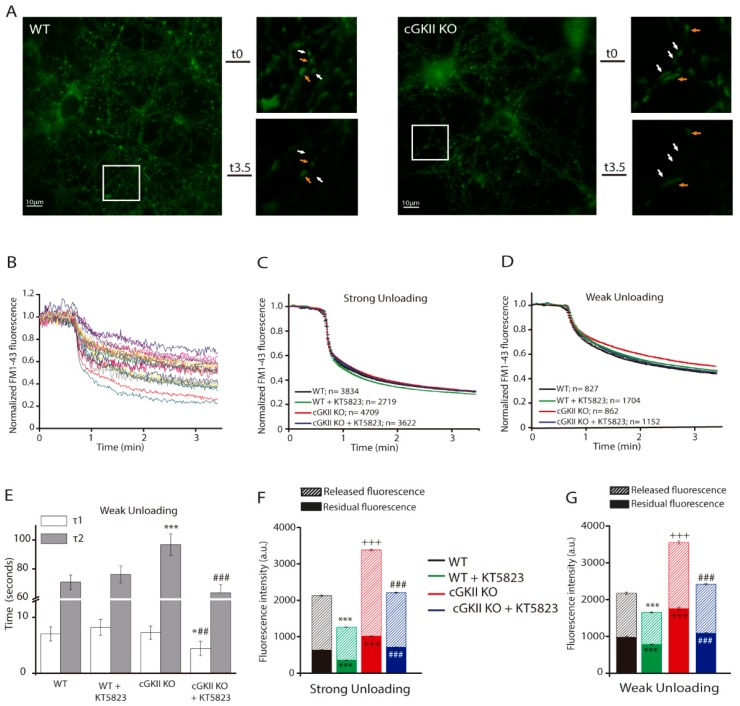

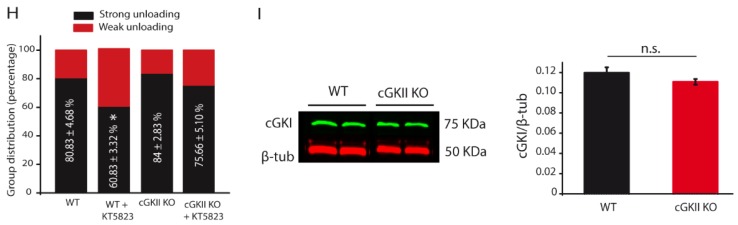
Efficient SV recycling in boutons from cGKII KO cells. (**A**) Fluorescence image of WT cells (left panels) loaded with FM1-43 (top amplified field) and unloaded (bottom amplified field), or those from cGKII KO mice (right panels). Bars: 10 μm. Amplified images of the boxed areas show group 1 (white arrows) and group 2 boutons (orange arrows); (**B**) Kinetics of FM1-43 unloading of 20 randomly selected synaptic boutons during sustained stimulation with 50 mM KCl; (**C**) Mean traces of boutons from WT cells that undergo strong FM1-43 unloading (*n* = 3834), from WT + KT5823 (1 μM) treated cells (*n =* 2719), from cGKII KO cells (*n* = 4709), and from cGKII KO + KT5823 (1 μM) treated cells (*n* = 3622). Fluorescent decay was adjusted to F = F_0_ + A_1_e^−t/τ1^ + A_2_e^−t/τ2^, where τ_1_ and τ_2_ are the time constants for dye unloading (τ_1WT_ = 4.76 ± 1.24 s and τ_2WT_ = 59.24 ± 5.31 s; τ_1WT + KT_ = 4.36 ± 0.90 s and τ_2WT + KT_ = 52.44 ± 5.92 s; τ_1KO_ = 3.30 ± 1.06 s and τ_2KO_ = 57.69 ± 4.27 s; τ_1KO + KT_ = 3.82 ± 1.06 s and τ_2KO + KT_ = 52.97 ± 5.93 s); (**D**) Mean traces of weak FM1-43 unloading boutons from WT cells (*n* = 827), from WT + KT5823 (1 μM) treated cells (*n* = 1704), from cGKII KO cells (*n* = 862), and from cGKII KO + KT5823 (1 μM) treated cells (*n* = 1152); (**E**) Graphical representation of the time constants for fluorescence decay of weak unloading boutons shown in panel D. Fluorescent decay was adjusted as indicated above, * *p* = 0.012 and *** *p* ≤ 0.001 represents significant differences against WT. ## *p* = 0.0068 and ### *p* ≤ 0.001 represents significant differences against cGKII KO without KT5823; (**F**) Comparison of FM1-43 uptake (total height of bars), released (striped bars) and residual staining (filled bars) in boutons corresponding to the strong unloading group from WT cells (*n* = 5 coverslips), WT cells incubated with KT5823 for 24 h (1 μM, *n* = 6 coverslips), cGKII KO cells (*n* = 7 coverslips) and cGKII KO cells incubated with KT5823 for 24 h (1 μM, *n* = 6 coverslips), expressed as the mean intensity (±SEM) from individual experiments (coverslips from three different cultures: *** *p* ≤ 0.001 initial fluorescence or residual fluorescence compared to WT or without KT5823; ### *p* ≤ 0.001 initial fluorescence or residual fluorescence compared to cGKII KO without KT5823; +++ *p* ≤ 0.001 initial fluorescence or residual fluorescence comparing WT vs. cGKII KO; (**G**) Comparison of FM1-43 uptake (total height of bars), release (striped bars) and residual staining (filled bars) in boutons corresponding to the weak unloading group from the same coverslips as panel (**F**); (**H**) Distribution of boutons as strong or weak unloading groups, expressed as the mean percentage (±SEM) from individual experiments (*n* = 5–7 coverslips; * *p* = 0.0336, one way ANOVA); (**I**) Western blot analysis of cGKI amount in extracts from cerebellar granule cells obtained from cGKII KO and WT mice.

Cerebellar granule cells in vivo receive strong and sustained stimulation trough mossy fiber (MF) terminals that can fire at over 200 Hz for sustained periods [19]. Because a very strong stimulus is necessary to mobilize the recycling pool in cerebellar granule cells and chemical stimulation provides such a strong stimulation [19], stimulation with 50 mM K^+^ was used to visualize synaptic vesicle recycling in mice cerebellar granule cells. We examined the ability of these cells to incorporate the fluorescent styryl dye FM1-43 into synaptic vesicles by stimulating them in the presence of the dye ([20] Figure 1A, whole field and upper panels of the amplified field) and, after washing the excess of dye, we assessed their capacity to unload the dye upon another round of stimulation (Figure 1A, bottom panels of the amplified field). Stimulation in the presence of 1.33 mM external Ca^2+^ resulted in a biphasic profile of dye unloading [21,22] and provokes distinct degree of discharge in individual boutons (Figure 1B). As published before [3,18,23], we segregated the boutons into two populations on the basis of the extent of unloading during the first min of stimulation, the strong unloading group (those released >40% of the initial fluorescence: Figure 1C) and the weak unloading group (those released <40% of the initial fluorescence: Figure 1D), and analyze the different parameters in them. The lack of cGKII or preincubation with the cGK inhibitor did not alter the kinetic profile of strong unloading boutons, which displayed a similar extent of dye release and unloading velocity with similar time constants (Figure 1C). However, the kinetic profile of weak unloading boutons from cGKII KO cells differed significantly showing a slower second component (Figure 1D,E). Moreover, the magnitude of dye loss after 2.5 min of KCl perfusion was about 20% lower in these boutons (Figure 1D). When cells were incubated with KT5823 no changes in the time constants were observed in WT boutons, but they were significantly reduced in cGKII KO boutons (Figure 1E).

The initial fluorescence, after washing the excess dye, reflects the amount of retrieved membrane during the loading stimulus, and it was higher in the two groups of boutons from cGKII KO cells than those of wild type cells (Figure 1F,G). Interestingly, the FM1-43 taken up by both groups of boutons from either WT or cGKII KO cells was significantly reduced when they had been incubated with the specific inhibitor for cGKs, KT5823. These results indicate that in these cells cGKI is also involved in the regulation of vesicle cycle in all the boutons and whereas the lack of cGKII increased the accumulated dye the inhibition of cGKI did the opposite. The majority of the boutons belonged to the strong unloading group in cells from both wild and cGKII KO mice (Figure 1H). When KT5823 (1 µM) was administered 24 h before the experiment, the percentage of strong unloading boutons significantly decreased in WT cells while it remained unchanged in the cGKII KO cells. The analysis of cGKI amount in cells obtained from either WT or cGKII KO mice revealed similar levels of protein (Figure 1I).

### 2.2. Electron Microscopy Reveals Differences between Synapses from Wild Type and cGKII KO Mice

**Figure 2:**
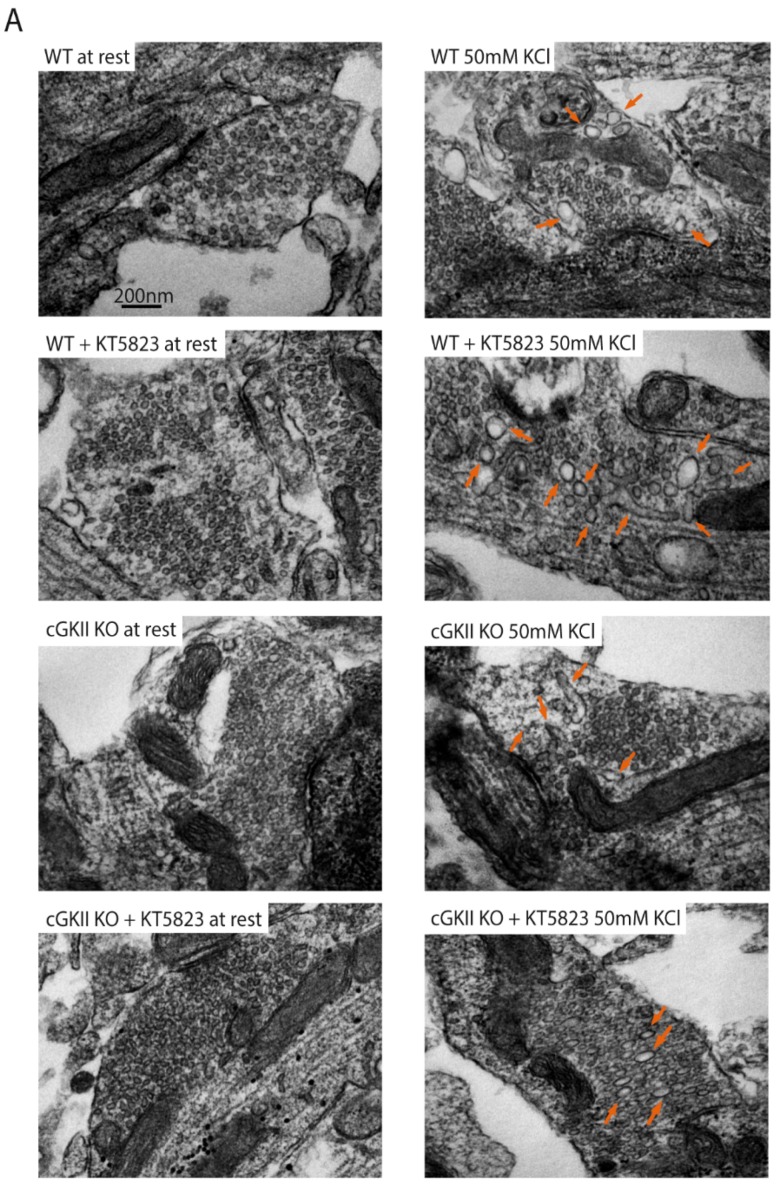

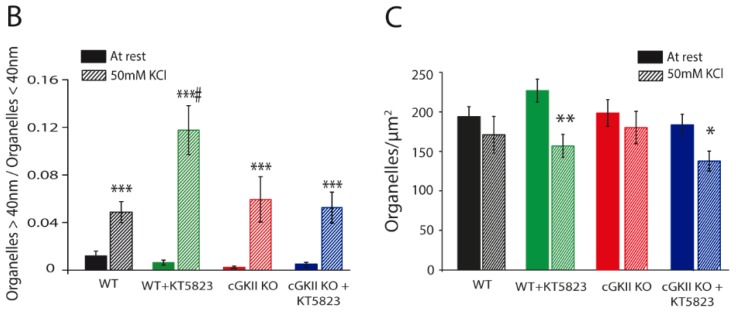
Morphological changes in nerve terminals from WT or cGKII KO cells at rest, or after strong and sustained stimulation. Cells were stimulated (50 mM KCl, 3 min) to mimic FM1-43 loading or maintained at rest. (**A**) Electron micrographs of cerebellar granule cells from WT cells, WT cells treated with KT5823, cGKII KO cells and cGKII KO cells treated with KT5823 at rest or after stimulation. Orange arrows show endosomal structures; (**B**) Ratio of organelles >40 nm/organelles <40 nm at rest or after stimulation: # Represents significant differences respect to the control and * the significance between the basal and stimulated cells in each condition. The data are the mean (±SEM) of WT AZs (at rest, *n* = 30), stimulated WT AZs (*n* = 38, *** *p* = 0.0001, Mann–Whitney), WT + KT AZs at rest (*n* = 29), stimulated WT + KT AZs (*n* = 34, *** *p* = 0.0001, # *p* = 0.014, Mann–Whitney), cGKII KO AZs at rest (*n* = 16), stimulated cGKII KO AZs (*n* = 14, *** *p* = 0.0001, Mann–Whitney), cGKII KO + KT at rest (*n* = 14), stimulated cGKII KO + KT AZs (*n* = 21, *** *p* = 0.0001, Mann–Whitney); (**C**) Organelle density at synapses. * represents the significance between the basal and stimulated cells in each condition. The data are the mean (±SEM) of WT AZs (at rest, *n* = 30), stimulated WT AZs (*n* = 38), WT + KT AZs at rest (*n* = 29), stimulated WT + KT AZs (*n* = 34, ** *p* = 0.0012, Mann–Whitney), cGKII KO AZs at rest (*n* = 16), stimulated cGKII KO AZs (*n* = 14), cGKII KO + KT at rest (*n* = 14), stimulated cGKII KO + KT AZs (*n* = 21, * *p* = 0.0222, Mann–Whitney).

Cerebellar cultured cells (7 DIV: WT, WT treated with KT5823, cGKII KO and cGKII KO treated with KT5823) and adult cerebellar tissue were analyzed by electron microscopy (EM), the cells having been maintained at rest or stimulated for 3 min in medium containing 50 mM KCl and incubated during 10 min in low KCl containing medium to mimic the FM1-43 dye loading step. The ratio of endosomes (organelles ≥ 40 nm) and small synaptic vesicles (sSVs), (organelles ≤ 40 nm) was calculated for each preparation. At rest, the endosome/sSV ratio was very low in the synapses of each of the four preparations (Figure 2A left panels and 2B), yet both the proportion of synapses with endosomes and the endosome/SV ratio increased after stimulation. These parameters were similar in WT and cGKII KO cells (Figure 2A right panels and 2B), yet a further increase was evident when WT cells were incubated with KT5823. Nonetheless, this was not the case in cGKII KO cells under the same conditions, where the increase in the ratio of endosome/sSV was not observed. The fact that treatment of WT synapses with KT5823 increases the endosome/vesicle relationship supports our interpretation that the lack or inhibition of cGKII hinders recycling because the formation of endosomes might be favored or their processing to form new vesicles slowed down in a subpopulation of boutons. Additionally, it explains the increase in the proportion of boutons with inefficient recycling manifested by a weak dye discharge as observed before. However, the ratio endosomes/SVs did not increase in the cGKII KO synapses, as it would be expected, although it explains why in these cells the majority of the boutons belonged to the strong unloading group likely due to the existence of a mechanism of compensation. The total number of organelles per synapse was assessed in the four preparations and the density (organelles/μm^2^) was similar in the four cell preparations in resting conditions (Figure 2C). However, a significant reduction of the organelle density was observed in stimulated WT and cGKII KO cells treated with KT5823.

**Figure 3:**
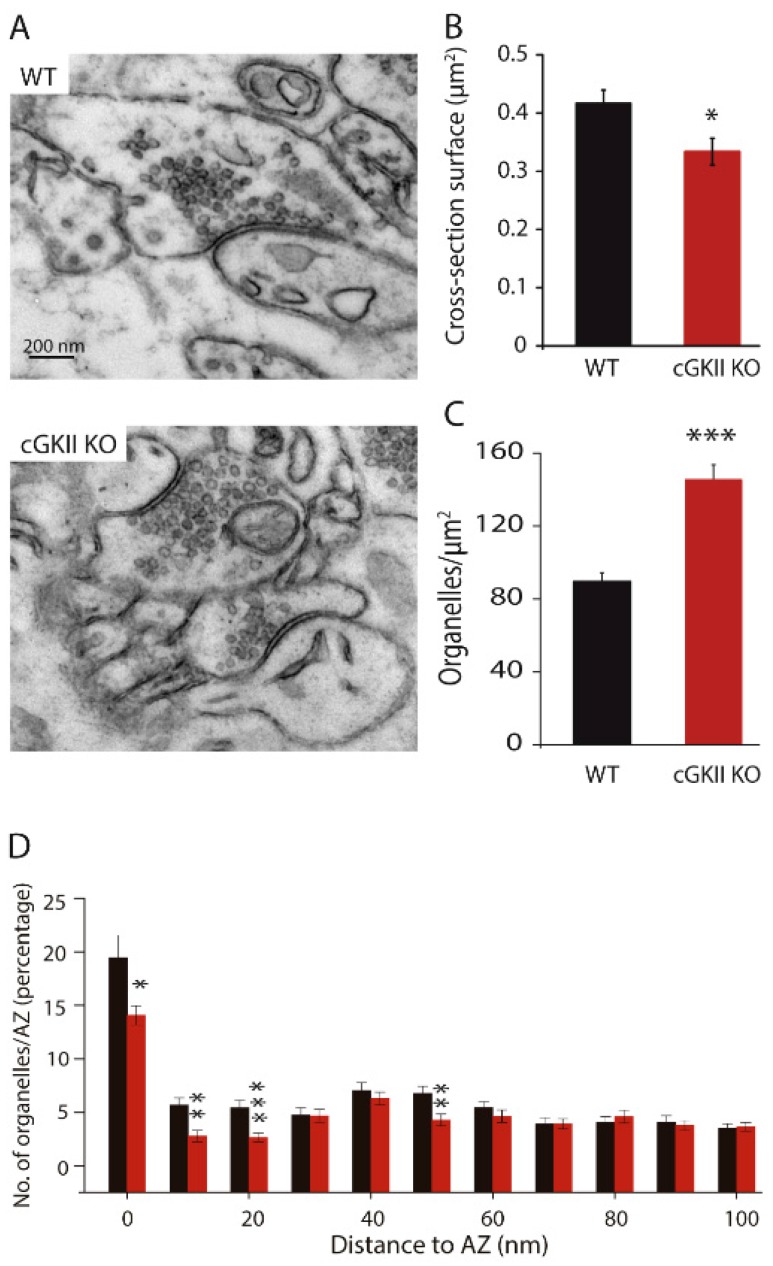
Morphological changes in nerve terminals from WT or cGKII KO adult cerebellum. (**A**) Electron micrographs of the cerebellar molecular layer of WT mice and cGKII KO mice. Scale bar = 200 nm; (**B**) Synapse cross-section surface expressed in μm^2^; (**C**) Organelle density at synapses; (**D**) Percentage of vesicles located in 10 nm bins at a distance from the active zone. Data plotted in panels B–D are mean values (±SEM) from 54 WT terminals and 41 cGKII KO terminals taken from two preparations each: * *p* = 0.025, ** *p* = 0.005; *** *p* = 0.0001, two-tailed *t*-test.

Although we did not find any structural difference between synapses of WT and cGKO cultured neurons, we wanted to analyze the synapses from cerebellar slices from WT and cGKII KO mice in order to see whether these synapses show any difference in adult animals (Figure 3A). Despite the fact that the analysis 2D performed in this study does not give information about the synapses’ volume, we found that the surface of synapses cross-section (Figure 3B) was larger in WT mice than in cGKII KO. By contrast, the density of organelles was higher in synapses from the cerebellum of cGKII KO mice (Figure 3C), an increase provoked by the reduced surface area coupled to an increase in SVs (WT, 35.92 ± 2.26 SVs/AZ; cGKII KO, 46.07 ± 3.25 SVs/AZ: *p* = 0.009, *t*-test). The distance of SVs to the AZ was calculated in 10 nm bins from the membrane and although a smaller proportion of SVs were associated with the AZ in cGKII KO synapses than in those from the WT, the mean number of SVs was quite similar (Figure 3D).

### 2.3. Altered Membrane Recycling in cGKII KO Neurons

To check the exo-endocytic cycle in neurons from WT and cGKII KO mice, as well as the effect of acute incubation with KT5823, cells were transfected with vGluT1-pH and a dual stimulation protocol was employed with KT5823 perfusion between the two stimuli. Cells were stimulated by KCl depolarization during either 15 s or 50 s followed by exposure to ammonium chloride (NH_4_Cl), as a measure of the total population of fluorescently labeled synaptic vesicles, giving the maximal fluorescence. The steady-state surface fraction of vGlut1-pH was determined before the first or second stimulus by examining the fractional change in fluorescence in response to NH_4_Cl after background subtraction [24].

**Figure 4:**
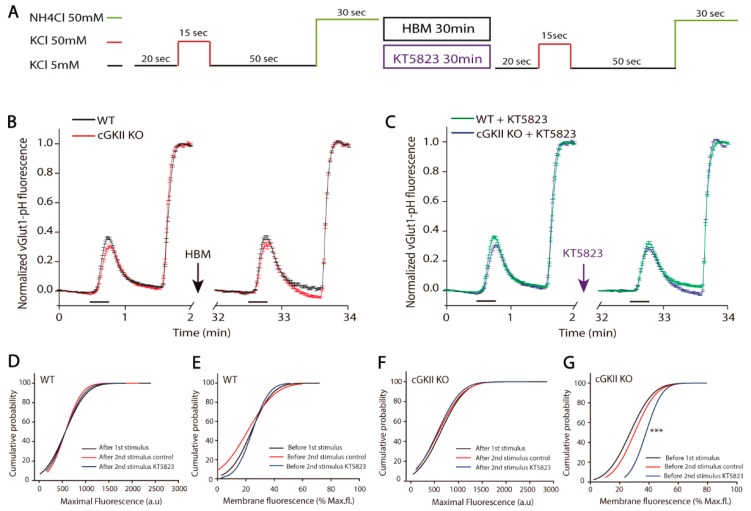
A dual stimulation protocol reveals important differences in the synaptic vesicle cycle in boutons from neurons lacking cGKII after KT5823 treatment. (**A**) Experimental protocol; (**B**) Mean traces of the KCl-elicited increase in vGluT1-phluorin fluorescence normalized to the NH_4_Cl-elicited fluorescence for all the boutons analyzed from WT (*n* = 167 boutons, 4 coverslips from three different cultures) or cGKII KO neurons (*n* = 114 boutons, 4 coverslips from three different cultures) perfused with HBM between the two stimuli; (**C**) Mean traces of the KCl-elicited increase in vGluT1-phluorin fluorescence normalized to the NH_4_Cl-elicited fluorescence for all the boutons analyzed from WT (*n* = 153 boutons, 3 coverslips from three different cultures) or cGKII KO neurons (*n* = 157 boutons, 3 coverslips from two different cultures) perfused with HBM containing KT5823 (1 μM) between the two stimuli; (**D**) Cumulative distribution of maximal fluorescence (obtained by a brief exposure to NH_4_Cl) after the first or the second stimulus in boutons from the WT cells represented in panel B and C; (**E**) Cumulative distribution of the membrane steady state fluorescence expressed relative to the maximum fluorescence before the first or the second stimulus ±KT5823 of boutons from the WT cells represented in panel B and C; (**F**) Cumulative distribution of the maximum fluorescence (obtained by a brief exposure to NH_4_Cl) after the first or the second stimulus in boutons from the cGKII KO cells represented in panel B and C; (**G**) Cumulative distribution of the membrane steady state fluorescence expressed relative to the maximum fluorescence before the first or the second stimulus of boutons from the cGKII KO cells represented in panel B and C (*** *p* = 0.0007, 2nd stimulus KT5823 vs. 1st stimulus, Mann–Whitney).

First, boutons were stimulated for 15 s, allowed to recover and then briefly perfused with NH_4_Cl. After a 30 min perfusion with hepes buffered medium (HBM) or HBM plus KT5823, the stimulation protocol was repeated (Figure 4A) and the mean of all the responding boutons was analyzed, small differences in the response of boutons from cGKII KO cells were found regarding the response of WT cells, particularly in terms of the response to the second stimulus (Figure 4B,C). The maximum fluorescence measured after each stimulus did not change in boutons from WT or cGKII KO cells in any experimental condition (Figure 4D,F). However, while the proportion of fluorescence at the membrane in WT boutons was similar in all conditions, in boutons from cGKII KO, cells perfused with KT5823 increased significantly (Figure 4E,G).

**Figure 5:**
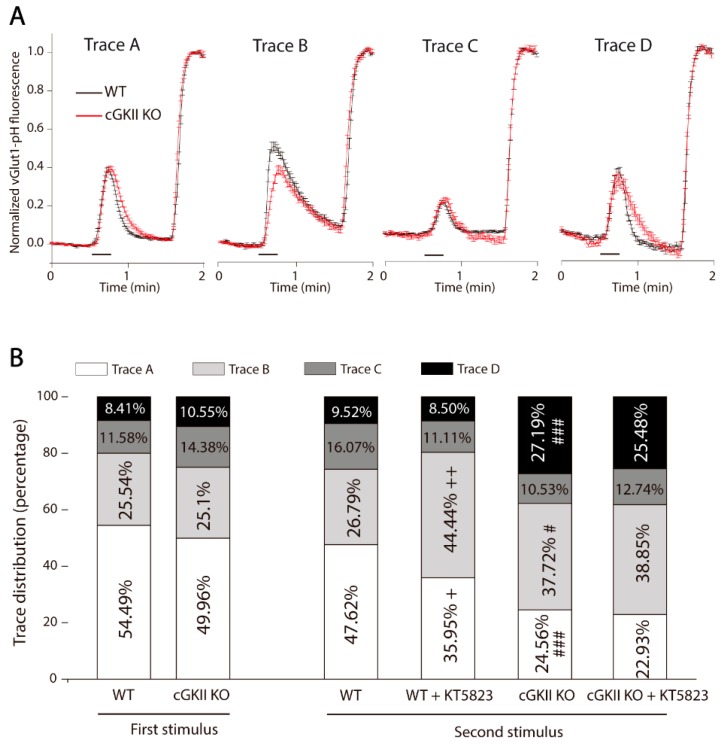
Heterogeneous traces of vGlut1-phluorin fluorescence recovery after strong and sustained stimulation (15 s, 50 mM KCl) in synaptic terminals from WT or cGKII KO cells. (**A**) Trace A: Mean traces of KCl-elicited vGluT1-phluorin fluorescence normalized to NH_4_Cl-elicited fluorescence in WT (*n* = 81 boutons) and cGKII KO neurons (*n* = 58 boutons). Trace B: Mean traces of KCl-elicited vGlut1-phluorin fluorescence normalized to NH_4_Cl-elicited fluorescence in WT (*n* = 38 boutons) and cGKII KO neurons (*n* = 34 boutons). Trace C: Mean traces of KCl-elicited vGluT1-phluorin fluorescence normalized to NH_4_Cl-elicited fluorescence in WT (*n* = 34 boutons) and cGKII KO neurons (*n* = 11 boutons). Trace D: Mean traces of KCl-elicited boutons) and cGKII KO neurons (*n* = 11 boutons); (**B**) Proportion of the four trace types found in WT and cGKII KO cells in response to the first stimulus or the second stimulus ±KT5823. + *p* = 0.035, ++ *p* = 0.0011 (WT 2nd stimulus ±KT5823). # *p* = 0.003, ### *p* = 0.00001 (cGKII KO 2nd stimulus vs. WT 2nd stimulus). The StacPac software was used to compare the percentages (two sample *t*-test to compare WT and cGKII KO, or a one sample *t*-test to compare responses to the 1st and 2nd stimulus).

The analysis of the responses of individual boutons showed a certain heterogeneity, and since these responses show the balance between exocytosis and endocytosis, this heterogeneity probably reflects the variations in size of the individual boutons, in their release probability [25,26], their calcium responses [27] or endocytic mechanisms [28,29,30]. The individual responses could be categorized as four different types called: A, B, C and D (Figure 5A and Table 1). Type A reflects a rapid increase in fluorescence followed by efficient recovery, with similar kinetics in boutons from WT and cGKII KO cells. In type B, the increase in fluorescence was a little slower in boutons from cGKII KO cells (Table 1) and the recovery, in both WT and cGKII KO boutons, was slightly slower than in A. In response type C, the increase in fluorescence was small and with slower recovery in boutons from cGKII KO cells than in WT boutons, and, in type D, there was a slightly higher basal fluorescence that dropped below the basal levels after recovering from the stimulus. The distribution of responses to the first stimulus among these four types was similar in boutons from WT and cGKII KO cells, and a similar distribution of responses was maintained following the second stimulus of boutons from WT cells perfused with HBM. However, the presence of KT5823 in the perfusion medium clearly altered this distribution, increasing the responses with a slowed down recovery that corresponded to the type B at the expense of the more rapid type A responses. In cGKII KO, cells that received a second stimulus also increased the proportion of boutons with responses with a slowed down recovery (type B and D) either in the absence or in presence of KT5823.

These findings suggest that the lack of cGKII or its inhibition slow down endocytosis in a subset of boutons when neurons receive repetitive stimulation, augmenting the proportion of boutons that undergo slow baseline fluorescence recovery. It is important to point out that the same stimulation protocol applied to rat cerebellar granule cells showed that the presence of KT5823 between the two stimuli did not augment the number of responses with a slow recovery of fluorescence (data not shown), further highlighting the differences between rat and mice cells.

**Figure 6:**
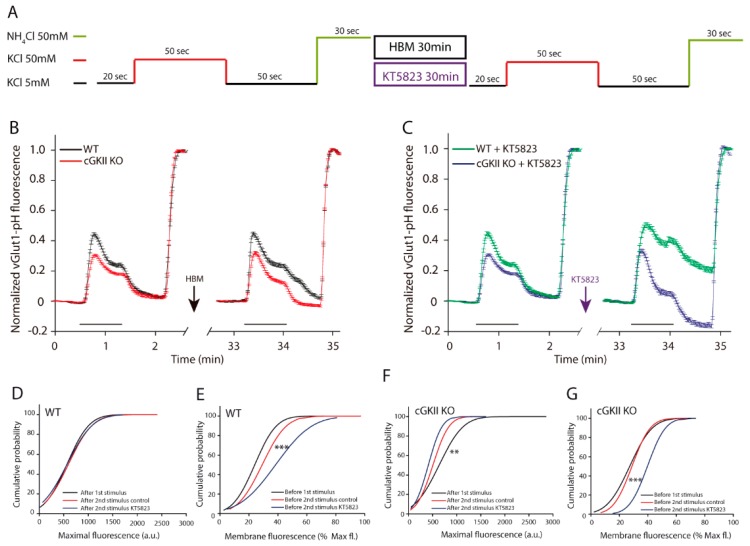
A double sustained stimulation protocol reveals important differences in the synaptic vesicle cycle of boutons from neurons lacking cGKII after KT5823 treatment: (**A**) Experimental protocol; (**B**) Mean traces of the increase in KCl-elicited vGluT1-phluorin fluorescence normalized to NH_4_Cl-elicited fluorescence for all the boutons analyzed from WT (*n* = 207 boutons, 4 coverslips from three different cultures) or cGKII KO neurons (*n* = 283 boutons, 5 coverslips from three different cultures) perfused with HBM between the two stimuli; (**C**) Mean traces of the increase in KCl-elicited vGluT1-phluorin fluorescence normalized to NH_4_Cl-elicited fluorescence for all the boutons analyzed from WT (*n* = 156 boutons, 4 coverslips from three different cultures) or cGKII KO neurons (*n* = 158 boutons, 4 coverslips from three different cultures) perfused with HBM containing KT5823 (1 μM) between the two stimuli; (**D**) Cumulative distribution of the maximal fluorescence (obtained by a brief exposition to NH_4_Cl) after the first or the second stimulus in boutons from the WT cells represented in panel B and C; (**E**) Cumulative distribution of the membrane steady state fluorescence expressed as a percentage of the maximal fluorescence before the first or the second stimulus ±KT5823 in boutons from the WT cells represented in panel B and C (*** *p* = 0.0056, 2nd stimulus vs. 1st stimulus, Mann–Whitney); (**F**) Cumulative distribution of the maximal fluorescence (obtained by a brief exposition to NH_4_Cl) after the first or the second stimulus in boutons from cGKII KO cells represented in panel B and C (** *p* = 0.0021, 2nd stimulus vs. 1st stimulus, Mann–Whitney); (**G**) Cumulative distribution of the steady state membrane fluorescence expressed as the percentage of the maximal fluorescence before the first or the second stimulus in boutons from cGKII KO cells represented in panel (**B**,**C**) (*** *p* = 0.0002, 2nd stimulus KT5823 vs. 2nd stimulus control or 1st stimulus, Kruskal–Wallis).

To examine how these boutons recycled their membranes after a longer stimulus, they were stimulated for 50 s, allowed to recover and then briefly perfused with NH_4_Cl. After a 30 min perfusion with HBM or HBM plus KT5823, the stimulation protocol was repeated (Figure 6A). When the two responses of individual boutons were normalized to the maximal fluorescence of each and the mean response was plotted, differences between the responses of the WT and cGKII KO synapses were found (Figure 6B,C). The responses of boutons from cGKII KO cells to the two stimuli were weaker than those of the WT cells, and the basal fluorescence was always recovered. By contrast, fluorescence was not completely recovered in WT cells after the second stimulus, being their recovery slower than after the first stimulus (Figure 6B). When KT5823 was perfused between the two stimuli, the basal fluorescence was not recovered in WT cells, even in the 50 s after the end of the stimulus. Conversely, the fluorescence of boutons from cGKII KO cells fell below the initial fluorescence values when the stimulus ended (Figure 6C). When the maximal fluorescence after the two stimuli and the steady-state fluorescence at the membrane before each stimuli (as the fraction of the maximal fluorescence) was assessed, the maximal fluorescence was maintained in WT cells after the two stimuli regardless of the presence of KT5823 (Figure 6D). However, there was stronger fluorescence at the membrane before the second stimulus than before the first one, an increase that was further enhanced in those boutons exposed to KT5823 (Figure 6E). It is important to note that this is the same effect as that observed in cGKII KO cells after the 15 s stimulus. Conversely, the maximal fluorescence in boutons from cGKII KO cells dropped after the second stimulus and this decrease was greater when the cells were exposed to KT5823 between the two stimuli (Figure 6F). Although the membrane fluorescence apparently remained unaltered, a clear increase was observed when it was expressed relative to the maximal fluorescence (Figure 6G).

**Figure 7:**
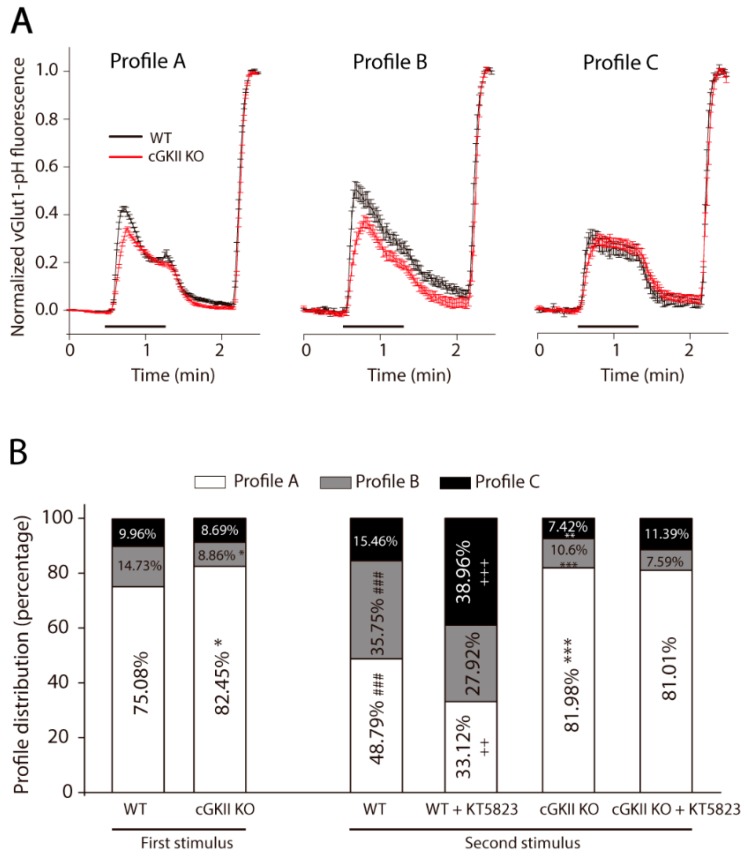
Heterogeneous profiles for vGlut1-phluorin fluorescence recovery after strong and sustained stimulation (50 s, 50 mM KCl) in synaptic terminals from WT or cGKII KO cells. (**A**) Profile A: Mean traces of KCl-elicited vGluT1-phluorin fluorescence normalized to NH_4_Cl-elicited fluorescence in WT (*n* = 159 boutons) and cGKII KO neurons (*n* = 235 boutons). Profile B: Mean traces of KCl-elicited vGluT1-phluorin fluorescence normalized to NH_4_Cl-elicited fluorescence in WT (*n* = 37 boutons) and cGKII KO neurons (*n* = 20 boutons). Profile C: Mean traces of KCl-elicited vGlut1-phluorin fluorescence normalized to NH_4_Cl-elicited fluorescence in WT (*n* = 18 boutons) and cGKII KO neurons (*n* = 28 boutons); (**B**) Proportions of the three types of profiles found in WT and cGKII KO cells in response to the first stimulus (WT *n* = 4 coverslips and cGKII KO *n* = 5 coverslips from three cultures; * *p* = 0.04) or the second stimulus ±KT5823 (WT *n* = 4 coverslips and cGKII KO *n* = 4 coverslips from three cultures). ### *p* = 0.0005 (WT 2nd vs. 1st stimulus). ++ *p* = 0.0022, +++ *p* < 0.0001 (WT 2nd stimulus ±KT5823). ** *p* = 0.0001, *** *p* < 0.0001 (cGKII KO 2nd stimulus vs. WT 2nd stimulus). The StacPac software was used to compare the percentages (two sample *t*-test to compare WT and cGKII KO percentages, or a one sample *t*-test to compare the responses to the 1st and 2nd stimulus).

The individual responses to the two stimuli of the boutons analyzed were also quite heterogeneous, although those of the WT and cGKII KO cells could be grouped into three types of profile (termed A, B and C), either perfused with HBM alone or HBM plus KT5823 (Figure 7A). In profile A, there was a rapid increase in fluorescence upon stimulation due to the exocytosis, followed by a decay in fluorescence due to the brief compensatory endocytosis until a steady-state was reached and maintained until the end of the stimulus. The time constants (τ) to reach the steady-state were similar for boutons from both WT and cGKII KO cells (τ_1_; Table 2). This steady-state was the consequence of the average of at least one hundred boutons (159 in WT and 235 in cGKII KO cells), because transitory increase of fluorescence was observed in individual responses suggesting that new exocytotic events took place. When the stimulus ended, the fluorescence decayed to basal levels with a similar time constant in both WT and cGKII KO boutons (τ_2_; Table 2). In profile B, there was a fast increase in fluorescence but the recovery was slow and with two components, the first lasting the duration of the stimulus and the second starting at the end of the stimulus. A steady-state was not reached in this type of response. In profile C, although the fluorescence increased rapidly, it did not reach the intensity of the other two responses, and it remained elevated while the stimulus persisted, thereafter decaying to basal levels. Thus, these three heterogeneous responses are mainly distinguished by the differences in fluorescence recovery, which mostly reflects differences in the endocytic mechanisms.

The response of the majority of the boutons from both WT and cGKII KO cells followed profile A (75.05% in WT cells and 82.45% in cGKII KO cells), and minimal differences were observed between the distributions of the responses to the first stimulus into the three profiles (Figure 7B). While in cGKII KO cells this distribution was the same for the first and second stimulus either in the absence or in the presence of KT5823, it was quite different in WT cells depending on the stimulus and on the presence or absence of KT5823 in the perfusion medium between the two stimuli (Figure 7B). In the WT cells, the response of the different boutons to the second stimulus changed notably, decreasing the proportion of those exhibited profile A in favor of those display profile B (Figure 7B). Furthermore, the presence of KT5823 drove a further drop in the proportion of type A responses (from 48.79% to 33.12%) while those following profile C augmented (15.46% to 38.96%).

## 3. Discussion

In this study, we identified several structural and functional differences in the synapses of mice cerebellar granule cells that lack cGKII respect to their WT controls, and also some processes related to the synaptic vesicle recycling that are regulated by cGKI. The FM1-43 dye taken up by synaptic vesicles after exocytosis enters the endocytotic pathways that again render vesicles competent for subsequent exocytosis. Thus, the extent by which FM1-43 staining is unloaded can be considered as a parameter of synaptic vesicle recycling at nerve terminals. Chemical stimulation was employed to mobilize the recycling pool and to incorporate the fluorescent styryl dye FM1-43 into SVs in cerebellar granule cells because it is necessary a strong stimulus [31]. This phenomenon is also observed in vivo as the synapses between mossy fibers (MFs) and granule cells are characterized by high rates of sustained vesicular release [32]. The fluorescence intensity was greater in cGKII boutons than in WT boutons suggesting that more vesicles became loaded with the dye, compatible with an increase in the recycling pool size when cGKII is absent [18]. Interestingly, the fluorescence taken up by the boutons from either WT or cGKII KO cells was significantly reduced by KT5823 pretreatment, uncovering a regulatory mechanism mediated by cGKI that is present in all the boutons from mice neurons and not previously observed in rat cells. The capacity of individual boutons to unload the dye upon another round of strong stimulation displayed a different efficiency in membrane recycling as described before [3,18,23]. This phenomenon together with the variability of the fluorescence accumulated in individual boutons, is a reflection of the heterogeneity of calcium responses observed in these boutons [27], since both the exocytosis and the different modes of endocytosis found in the central synapses are tightly regulated by calcium [28,29,30].

We have previously shown that in rat cerebellar granule cells exists a dynamin-independent FM1-43 uptake mediated by endosome-like structures formed during massive exocytosis that fail to recycle into release competent synaptic vesicles, at least in the time frame of our experiments, and that might contribute to boutons with weak unloading [23]. The lack of cGKII increased the proportion of these boutons in parallel to a higher endosome/SVs ratio in a subset of boutons [18]. An increase of the boutons with ineffective recycling and weak unloading and a greater endosome/SV ratio is also observed in WT mice cerebellar granule cells incubated with KT5823, which inhibits the two cGMP-dependent kinases. However, none of these phenomena were observed in cells from cGKII KO mice as it would had been expected, therefore suggesting that the lack of this protein has been compensated [17,33]. The fact that cerebellar granule cells express both cGKI and cGKII and that which share common substrates [34,35], might suggest some compensation by each other. However, this does not seem to be the case because the amount of cGKI was unaltered in cGKII deficient cells and the inhibition of cGKI in cGKII deficient cells did not alter the distribution of boutons in the strong and weak unloading groups and neither did the endosome/SVs ratio increase as in WT cells. These findings confirm the necessity of cGKII to efficiently recycle SVs following the massive exocytosis caused by a strong and sustained stimulation in a subset of boutons [18] and suggest the existence of a compensatory mechanism in cGKII KO cells. This mechanism would imply either a reduction in the rate of endosome formation by a greater contribution of clathrin-mediated endocytosis or, alternatively, a more efficient processing of the endosomes to SVs [36]. Although we have not analyzed possible substrates for cGKII that might underlay this mechanism, we could speculate with the idea that the lack of cGKII might result in an increased Akt activity [37], which in turns would inhibit the GSK3, the dynamin phosphorylation at Ser-774 and the activation of bulk endocytosis [38].

Despite the apparent compensatory mechanism to efficiently recycle SVs in cGKII deficient cells, when vGluT1-pH was used to follow the balance between the exocytosis and the endocytosis under strong stimulation several alterations were evident relative to the WT cells. These data confirmed the necessity of cGKII to maintain efficient membrane retrieval under strong and sustained stimulation, affecting the rate of recovering of vGluT1 and likely other vesicular cargos [24]. This conclusion is supported by the fact that pharmacological inhibition of cGKs with KT5823 in WT and cGKII KO cells renders similar results (with few exceptions) when a cell received two stimuli of 15 s. The analysis of the individual responses to the first stimulus in both WT or cGKII KO cells showed that, while the first stimulus elicited the same distribution of the responses in control and cGKII KO cells, the responses to the second stimulus differed in both cGKII KO and KT5823 treated WT cells, increasing the responses with a higher time constant for baseline recovery and, therefore, showing an impairment of the post-stimulus endocytosis in a subset of boutons due to the lack of cGKII or its inhibition that might cause a reduction of phosphatidylinositol-4,5-bisphosphate (PIP_2_) levels [10,13].

When the stimulus lasted 50 s, the averaged response of boutons from cGKII KO cells was weaker than that in the WT cells, regardless of how many stimuli preceded it or whether KT5823 was present. However, the major difference found was the decrease in the maximal fluorescence after the second stimulus, a phenomenon only observed in cGKII KO boutons and that was potentiated by KT5823. In conjunction with the significant reduction in SV density observed by EM, this result suggests that such a strong stimulus might cause the dispersion of SVs [39,40]. Thus, the inhibition of cGKI may favor SV dispersion or may impair their reclustering when the strong and extended stimulus desists. This effect is associated with the duration of the stimulus because the maximal fluorescence was unaltered when the stimulus duration was shorter.

Another result that deserves attention is that, after the second stimulus, the fluorescence of cGKII KO boutons decreases beyond baseline (independently of the duration of the stimulus). This fact is likely due to the enhanced fluorescence accumulated in the membrane before the second stimulus and a higher activation of endocytosis overshoot in these boutons [41].

These results demonstrate that neurons from cGKII deficient mice show many synaptic alterations suggesting that cGKII is important to maintain high-fidelity synaptic transmission in cerebellar granule cells under strong and sustained stimulation, as occurs in vivo [32], through the coordination of presynaptic and postsynaptic events [5,6,11]. The failure of this coordination might cause neurological disorders such as those observed in these mice, which show spatial memory defects, working memory impairments and increased anxiety-like traits [16,42]. However, this mice also showed an enhanced motor co-ordination [16], compatible with the more efficient recycling described here likely due to the existence of a compensatory mechanism. Although all together these data highlight the importance of cGKII in coordinating the activity in different brain areas in order to ensure normal learning and memory, further work is necessary to clarify if the synaptic alterations showed in cGKII lacking cerebelar neurons occurs in other synapses and underlay the neurological deficits of these mice.

## 4. Materials and Methods

### 4.1. Cell Culture

All procedures with animals were conducted under the ethical guidelines for animal experiments and the regulations established in the European Council Directive 2010/63/EU. The experimental protocols were approved by the Commission for Animal Experimentation at the Complutense University (CEA-UCM 4/2012, 7 February 2012) and all efforts were made to minimize the number of animals used and their suffering. Mice were housed with their mother under controlled temperature and lighting conditions with food and water provided ad libitum until postnatal day 5 (P5) in the Animal Facility of Universidad Complutense de Madrid. Cells were dissociated from the cerebellum of homozygous cGKII^−/−^ [43] and wild-type P5 mouse pups of either sex (129S/v strain: [44]) using the papain dissociation system, as described previously [45]. The genotypes of the wild-type and cGKII^−/−^ mice were identified by polymerase chain reaction analysis of ear DNA biopsies, using the following primers to amplify both the complete and interrupted cGKII gene: AV3R (5′-ATT AAG GGC CAG CTC ATT CC-3′), AV9R (5′-CTG CTT AAT GAC GTA GCT GCC-3′) and E2FB-AV (5′-GGT GAA GTT TTA GGT GAA ACC AAG-3′). The predicted size of the amplified fragments for the wild type and cGKII^−/−^ mice were 275 and 450 bp, respectively, and both fragments were detected in heterozygous mice. The primary cerebellar cells isolated were diluted in Neurobasal A supplemented with B27 (Life Technologies, Alcobendas, Madrid, Spain), 20 mM KCl, 0.5 mM glutamine and a stabilized antibiotic antimycotic solution (Sigma-Aldrich, Tres Cantos, Madrid, Spain). The cells were seeded onto poly-l-lysine coated coverslips at a density of 3 × 10^5^ cells/coverslip, and the cultures were maintained in a humidified incubator at 37 °C in an atmosphere of 5% CO_2_. After 24 h in culture, cytosine-α-d-arabinofuranoside (10 μM: Sigma-Aldrich) was added to restrict glial cell growth and the cultures were then routinely used in experiments between 7 and 9 days in vitro (DIV).

### 4.2. Cell Transfection

Dissociated neurons were electroporated with the chicken actin pCAGGs vector (1 μg) containing the vGlut1-pHluorin construct using an Amaxa^TM^ Nucleofector II device and the Rat Neuron Amaxa Nucleofector Kit (Lonza, Basel, Switzerland), according to the manufacturer’s instructions (program O-003). The cells were then grown as described previously until 7-8 DIV [3].

### 4.3. Western Blotting

Proteins from 7 DIV cells were separated on 8% sodium dodecyl sulphate-polyacrylamide gels and transferred to nitrocellulose membranes (Hybond ECL: GE Healthcare Life Sciences, Madrid, Spain). The membranes were probed with the primary antibodies raised against cGKI (1:1000, RRID: AB_593072); β-tubulin (1:2000, RRID: AB_477556). After several washes, the membranes were incubated with the corresponding IRD-labeled secondary antibodies (LI-COR Biosciences, Alcobendas, Madrid, Spain): goat polyclonal anti mouse IRD 680 (RRID: AB_621840) or goat polyclonal anti rabbit IRD 800 (RRID: AB_621843). The membranes were scanned in an Odyssey Infrared imaging system, and the immunolabeling of proteins was compared by densitometry and quantified using Odyssey 2.0 software. The data were normalized to the β-tubulin signal to account for loading differences.

### 4.4. Vesicle Recycling (FM1-43)

Recycled SVs were labeled with the cationic styrylpyridinium dye FM 1–43 (PubChem CID: 6508724; Molecular Probes, Invitrogen, Madrid, Spain) as described previously [3,23]. Briefly, the cells were incubated for 10 min in a calcium free buffer (140 mM NaCl, 5 mM KCl, 5 mM NaHCO_3_, 1.2 mM NaH_2_PO_4_, 1 mM MgCl_2_, 10 mM glucose, 10 mM HEPES, pH = 7.4), and they were then incubated for 3 min with FM1-43 dye (10 μM) in high potassium buffer (95 mM NaCl, 50 mM KCl, 5 mM NaHCO_3_, 1.2 mM NaH_2_PO_4_, 1 mM MgCl_2_, 1.33 mM CaCl_2_, 10 mM glucose, 10 mM HEPES, pH = 7.4). The coverslips were mounted in a rapid-switching, RC-20 laminar-flow perfusion chamber (volume: 36 μL; Warner instruments, Hamden, CT, USA) and a PH-5 platform (Warner instruments) on the stage of a Nikon Eclipse TE2000-S inverted epifluorescence microscope, and they were continuously perfused (1 mL/min) using a VC6 perfusion system (Warner instruments). The cells were then washed for 10 min with a calcium-free low potassium buffer to remove the surface-bound dye and baseline measurements were acquired over 30 s. Subsequently, the cells were stimulated for 3 min with high potassium medium to produce dye unloading. Temperature was clamped at 37 °C with a TC-344B temperature controlling system (Warner instruments) to minimize the effects of temperature fluctuations. Images were acquired at a rate of 1 Hz using an iXon^EM^ + EMCCD camera (iXon^EM^ + DU885, Andor Technology, Belfast, UK) and fluorescence emission was recorded through a Nikon CFI Plan Apo VC 60X Oil objective 1.4 (NA). Excitation was provided by a 479 nm monochromator and the light emitted was collected through a fluorescein isothiocyanate (FITC) filter.

### 4.5. Analysis of FM1-43

Different fields were selected randomly and individual synaptic boutons were analyzed as described previously [23]. Regions of interest (ROIs) were identified with Igor Pro software (version 6.3.7.2, WaveMetrics, Inc., Portland, OR, USA; RRID: SCR_000325) to define the synaptic boutons according to the automated method described elsewhere [46], employing a minimum quality criterion for analysis of 0.1. To determine the extent of dye release, the mean background fluorescence of several cell free regions was subtracted and the values were normalized to the initial fluorescence (F/F0). When the rate of dye release was calculated, we subtracted the remaining fluorescence of each bouton at the end of the stimulation period. Dye release responses were heterogeneous; however, two main subpopulations of synaptic boutons were distinguished [23].

### 4.6. vGlut1-pHluorin (vGlut1-pH) Imaging

To analyze exocytosis (reflected by an increase in fluorescence) followed by endocytosis (fluorescence decay), we used a direct optical presynaptic readout based on the pH-sensitive GFP pHluorin [47] tagged to the luminal domain of the vesicular glutamate transporter. The total vesicle population was visualized by the subsequent application of NH_4_Cl saline. The decay phase of the vGlut1-pH signal reflects the rate of synaptic vesicle endocytosis and re-acidification that follows vesicle retrieval [48,49]. Endocytosis time constants with single exponential decays were fitted using OriginPro (OriginLab, RRID: SCR_014212), with a temporal offset for reacidification of ~5 s [50]. Images were acquired at a rate of 4 Hz and averaged into one single frame per second in order to enhance the signal-to-noise ratio, resulting in an actual readout of 1 Hz.

### 4.7. Electron Microscopy (EM)

The effect of the different treatments or experimental conditions on the distribution or recycling of SVs within pre-synaptic terminals was visualized in cultured cerebellar granule cells. The cells were maintained under basal conditions or stimulated for 3 min with 50 mM KCl in HBM (HEPES-buffered medium), and they were then maintained at rest for 10 min in 5 mM KCl in HBM, emulating our protocol for the loading and washing of the FM1-43 dye. Then, cells were fixed and processed as previously described [18]. Ultrathin sections (70 nm) were then stained routinely with uranyl acetate and lead citrate, and images were obtained on a JEOL 1200 EX II transmission electron microscope (TEM) (JEOL USA, Inc., Peabody, MA, USA). Synapses were identified by the clusters of synaptic vesicles in close proximity to a membrane with docked (from a morphological point of view) vesicles. Synapses were also considered to have an apposed postsynaptic cell or dendrite.

In addition, to analyze the ultrastructure of adult synapses, cerebellum slices (325 μm) were fixed with 3.5% glutaraldehyde in sodium phosphate buffer (0.1 M, pH 7.3) for 45 min at 37 °C. These slices were then left for 30 min at room temperature, stored for 20 h at 4 °C and then rinsed several times with abundant 0.1 M phosphate buffer [51]. Sections were post-fixed in 1% OsO_4_ for 1 h at room temperature, rinsed with distilled water and dehydrated in ethanol. After dehydration, the sections were embedded in Spurr (Spurr Embedding Kit, TAAB Laboratories Equipment Ltd., Berkshire, UK) and polymerized prior to obtaining ultrathin sections (70 nm), which were routinely stained with uranyl acetate and lead citrate. Images obtained on a JEOL 1200 EX II TEM were analyzed with the aid of ImageJ software (RRID: SCR_003070): the diameter of the SVs and the rest of the organelles in the bouton; the number of each organelle type within the synapse cross-section; and the cross-section surface. As such, the relative percentage of SVs per AZ was calculated in 10 nm bins from the AZ membrane, using the outer membrane of the SVs and the inner layer of the AZ plasma membrane as reference points. The total number of vesicles per cross-section of synaptic terminal was also determined.

### 4.8. KT5823 Treatment

For FM1-43 experiments and TEM, cells were incubated with KT5823 (PubChem CID: 3843; 1 μM) in culture medium for 24 h before the experiment. For vGluT1-pH experiments, cells were incubated (by perfusion) with HBM containing KT5823 (1 μM) for 30 min between the two stimuli. The concentration of KT5823 was selected based on previous reports [52,53,54,55].

### 4.9. Statistical Analyses

The data were analyzed with Statgraphic Centurion XVII (RRID: SCR_015248), OriginPro 8.0 (RRID: SCR_014212), SigmaPlot 11 (RRID: SCR_003210) or StatPac statistical software. The Shapiro–Wilk test was used to check for normal distribution data. For not normally distributed data non-parametric test were applied: Mann–Whitney or Kolmogorov-Smirnov test for the comparison of two independent groups and Kruskal–Wallis test for the comparison of several independent groups. For normally distributed data, the comparison of means was performed with the *t*-test (two groups) or using one-way ANOVA followed by a Bonferroni test (several independent groups). In all analyses, a family wise 95% confidence level (*p* < 0.05) was applied: “*n*” indicates the number of individual experiments performed using at least two different cell cultures or the number of specimens analyzed as indicated (ROIs, boutons, etc.).

## Figures and Tables

**Table 1:** Time constants of exocytosis and endocytosis for the different traces in response to brief stimulation.

	Wild Type		cGKII KO	
	k (s)	τ (s)	k (s)	τ (s)
**Trace A**	6.21 ± 0.51	7.59 ± 0.31	5.99 ± 0.30	10.92 ± 0.55
**Trace B**	2.80 ± 0.13	22.07 ± 0.93	6.66 ± 0.23 *p* < 0.0001	19.13 ± 0.83
**Trace C**	7.30 ± 0.73	6.93 ± 0.62	7.51 ± 0.74	15.44 ± 2.61 *p* = 0.0001
**Trace D**	5.84 ± 0.15	7.21 ± 0.36	5.69 ± 0.18	17.50 ± 0.76 *p* < 0.0001

Cells were stimulated with 50 mM KCl for 15 s and the increase in fluorescence recorded was adjusted to (F) = F_max_ × t/(k + t), where k is the time necessary to reach half maximal fluorescence. Florescent decay was adjusted to F = F_0_ + Ae^−t/τ^, where τ is the time constant for the basal recovery. The data are the mean ± SEM from individual experiments control (*n* = 10, wild type and *n* = 8, cGKII KO cells).

**Table 2:** Time constants of exocytosis and endocytosis for the different response profiles after strong and sustained stimulation.

	Wild Type			cGKII KO		
	k (s)	τ_1_ (s)	τ_2_ (s)	k (s)	τ_1_ (s)	τ_2_ (s)
**Profile A**	5.42 ± 0.23	14.74 ± 2.40	10.66 ± 0.40	5.58 ± 0.16	16.94 ± 1.54	10.22 ± 0.70
**Profile B**	5.07 ± 0.20	57.29 ± 7.16	22.21 ± 1.52	5.17 ± 0.13	38.05 ± 8.70 (*p* = 0.00511)	10.69 ± 0.59 (*p* < 0.0001)
**Profile C**	5.13 ± 0.19	18.76 ± 1.06		5.75 ± 0.12	11.76 ± 0.68 (*p* < 0.0001)	

Cells were stimulated with 50 mM KCl for 50 s and the increase in fluorescence recorded was adjusted to (F) = F_max_ × t/(k + t), where k is the time necessary to reach half maximal fluorescence. Florescent decay was adjusted to F = F_0_ + A_1_e^−t/τ1^ + A_2_e^−t/τ2^ where τ**_1_** and τ**_2_** are the time constants.

## Abbreviations

ADBE	Activity-dependent bulk endocytosis
Akt	Phosphoinositide 3-kinase activated serine/threonine kinase
ADBE	Activity-dependent bulk endocytosis
AMPAR	α-amino-3-hydroxy-5-methyl-4-isoxazolepropionic acid receptor
AP-1/AP-3	Adaptor protein 1 and 3 complex
AP-2	Adaptor protein 2 complex
AP3B2	AP-3 complex beta-2 subunit
AZ	Sctive zone
cGKI	Cyclic GMP dependent protein kinase type I
cGKII	Cyclic GMP dependent protein kinase type II
DIV	Days in vitro
EM	Electron Microscopy
FM1-43	*N*-(3-Triethylammoniumpropyl)-4-(4-(Dibutylamino) Styryl) Pyridinium Dibromide
GSK3	Glycogen synthase kinase 3
HBM	Hepes buffered medium
KO	Knock out
N.D	No difference
NMDAR	*N*-Methyl-d-aspartic acid receptor
P7	Seven days postnatal
pCAGGs	CAG promoter is a strong synthetic promoter frequently used to drive high levels of gene expression in mammalian expression vectors
sGC	Soluble guanylyl cyclase
SV	Synaptic vesicle
vGluT1-pH	pH-sensitive GFP tagged to the luminal domain of the vesicular glutamate transporter 1
